# A Bayesian Multi-Layered Record Linkage Procedure to Analyze Functional Status of Medicare Patients with Traumatic Brain Injury

**DOI:** 10.23889/ijpds.v9i5.2550

**Published:** 2024-09-18

**Authors:** Roee Gutman, Kali Thomas, Mingyang Shan

**Affiliations:** 1 Brown University; 2 Johns Hopkins School of Nursing; 3 Eli Lilly and Company

Understanding associations between injury severity and post-acute care recovery for older adults with traumatic brain injury (TBI) is crucial to improving care. Estimating these associations requires information on patients’ injury, demographics, and long-term health outcomes. This data is despersed across two different datasets: Medicare claims data and the National Trauma Data Bank (NTDB). Because of privacy regulations, unique identifiers are not available to link records across these two data sets. Record linkage methods identify records that represent the same patient across data sets in the absence of unique identifiers. With a large number of records, these methods may result in many false links. Health providers are a natural grouping scheme for patients, because only records that receive care from the same provider can represent the same patient. In some cases, providers are defined within each data set, but they are not uniquely identified across data sets. We propose a Bayesian record linkage procedure that simultaneously links providers and patients. The procedure improves the accuracy of the estimated links compared to current methods. We implement the proposed method to examine the associations between functional status assessments and TBI patients' ability to be independent following their injury by linking records from Medicare enrollment records and the NTDB.

